# How Does Leader Narcissism Influence Employee Voice: The Attribution of Leader Impression Management and Leader-Member Exchange

**DOI:** 10.3390/ijerph16101819

**Published:** 2019-05-22

**Authors:** Shudi Liao, Xingchi Zhou, Zhiwen Guo, Zhifei Li

**Affiliations:** 1 Business School, Hubei University, Wuhan 430062, Hubei, China; shudiliao@hubu.edu.cn (S.L.); guozhiwen@hubu.edu.cn (Z.G.); 2 School of Management, Wuhan Textile University, Wuhan 430073, Hubei, China

**Keywords:** perceived leader narcissism, employee voice, leader impression management, leader-member exchange

## Abstract

Recently, the influence of leader’s personality traits on employee behavior has become an emerging research area. Leaders play a crucial role in any organization because team members look up to them for policy and behavioral guidelines. Based on the social exchange theory, this study is focused on the relationship of employee-perceived leader narcissism and employee voice behavior. Through the analysis of 239 questionnaires, we find that leader narcissism has a significant influence on the motivation of leadership impression management. The narcissistic leader uses impression management that is more likely to have self- serving purpose rather than pro-social motivation. This motivation impacts leader-member exchange (LMX) quality which influences employee voice behavior. This study has significant theoretical and practical implications as it is the first study that empirically verifies the stated relationship in this under-researched area.

## 1. Introduction

Employee voice behavior is defined as promoted behavior that employee intends to adopt for the purpose of improving an organization’s standard procedures and performance, as well as facing challenges and making innovative suggestions with colleagues or leaders spontaneously [1,2,3]. Studies have shown that employee voice behavior plays an important role of decision making in leadership, which is one of the main factors that promote the development of the organization [4,5]. A leader needs to pay more attention to employee voice behavior in order to diagnose and prevent problems in today’s increasingly volatile market [6]. Scholars should focus on how to promote employee voice behavior in an organization, which has become an emerging research topic these days. In these studies, leadership style and behavior as well as personality traits are some of the key factors that influence employee voice behavior [2].

However, although some scholars have discussed the relationship between leaders’ personality traits and employee voice behavior, most of these researches focus on the positive side of leadership behaviors and characteristics, such as transformational leadership, charismatic leadership, and leader openness, etc. [2,7,8]. Recently, a growing body of literature has paid attention to the negative side of or destructive leadership styles, such as leader narcissism, or narcissistic leadership. Leader narcissism is a unique personality trait that exists in intrinsic personality and expresses in behaviors, which has an important influence on employee behaviors in an organizational context. For example, narcissistic individuals often fail to learn from experience due to their overconfidence [9]; narcissistic leaders are usually more aggressive on people who are considered offensive to them [10] and narcissistic individuals are found to be more related to counterproductive work behaviors [11]. However, few studies have focused on the relationship between leader narcissism and employee voice behavior. Liu and his colleagues presented empirical evidence that leader narcissism would affect follower’s voice behavior [12]. But there is are no studies exploring the relations between these two constructs from a follower’s perspective. In this paper, we aim to explore the mechanism between leader narcissism and employee voice from the perspective of follower perception of leader impression management. 

Impression management refers to a series of interactive behaviors that allow individuals to gain and maintain positive images that others hold of them. By consciously or unconsciously employing these behaviors, individuals are able to “conform to social norms, avoid blame or gain credit, maintain or enhance their self-concept, and strategically wield power and social influence” [13]. Research has shown that individuals always encounter each other in the process of impression management by interpersonal communication. They establish their self-image and connect it to their relationships with the people around them [14]. Similarly, individuals determine their behavioral strategy through the perception of their impression on others. In the same way the impression management of leadership would affect the relationship between leader and employee, and in turn influence on employees’ voice behavior. Nonetheless, with different personality traits of leadership, it has different motivation through impression management. Employees may react differently depending on types of motives they detected from their leaders. Because narcissistic leaders are keen on their self-interests, they barely pay attention to others’ needs [15]. Therefore, narcissistic leaders are more likely to do impression management because of the self- serving motivation, but not the altruistic motives for others.

In organizations, leaders and their followers will develop and maintain a certain type of exchange relationships over time which is known as leader-member exchange (LMX) [16]. Scholars found that a high-quality LMX relationship is important both for the success of leaders and followers [17]. Unlike impression management behavior which only focused on personal interest and success, the high-quality LMX relationships are based on mutual trust, respect, liking and obligation, and usually are reciprocal relationships [16]. In high-quality LMX relationships, leaders support their followers beyond general expectation, so as the followers are more engaged and proactive in their work. Thus, it is very likely that the leader-member exchange quality (LMX quality) between employee and leader may also influence on employee voice behavior. 

In this present study, we explore the relationship between leader narcissism and employee voice behavior by using impression management and social exchange theory to reveal the mechanism. This research contributes to the existing literature in the following aspects: First, leader narcissism and employee voice behavior evolve in two relatively separate research fields. Although some scholars tested the relationship between leader narcissism and employee voice behavior [12], there is still a need for further exploring the mechanism between these constructs for different theoretical perspectives. Thus, we look into the mechanism by introducing the impression management and social exchange perspective. Secondly, according to social exchange theory, we reveal the mediation mechanism between leader narcissism and employee voice behavior. The previous studies on leadership and employee voice behavior focus on the perspectives of “risk” and “benefit” to reveal employee voice behavior. However, this paper is based on social exchange theory to depict the relationship between leader narcissism and employee voice behavior via motive attribution in exchange process which is an important complement of current research. In addition, the concept of narcissistic leader has caught the attention of managers and scholars when it was first brought up. However, its impacts and mechanism have barely been studied. Hence, some scholars point out that the relationship among the narcissistic leader and some other organization outcome factors such as employee creativity and voice need further research [15].

## 2. Literature Review and Hypotheses

The concept “narcissism” comes from the word “narcissus”, which represents three meanings: (1) inflated self-concept; (2) Interpersonal exploitation; (3) Inordinate need for tribute from others [18]. Narcissistic individuals tend to be overconfident, and have a grandiose sense of self-importance and entitlement [19]. When a leader has narcissistic personality trait, it expresses in four ways: (1) Charisma; shows better social communication skills, also have the foresight and attractive appearance [15,20]. (2) Egoistic motives; often driven by self- serving needs rather than motives of organizational interests. (3) Deceptive motives; use spurious tools such as impression management or mendacious care to mobilize others for self-interests [21]. (4) Knowledge inhibition; sensitive to others’ criticism, eager to get praise and hostile to negative feedback [22]. Therefore, narcissistic leaders have made followers puzzled in some ways, are able to arouse the initiatives of followers but also have the dark side.

Narcissism has existed in team leaders which happen to be a common personality trait. Numerous CEOs (i.e., Chief Executive Officer) have been identified as narcissists, such as Jack Welch, Steve Jobs, etc. Maccoby lists that narcissistic individuals have two strengths: they are visionaries which can influence and inspire a lot of followers [23]. As visionaries, narcissistic leaders always try hard to make their own future and tend to be more aggressive and creative in work because they are inspired by the glory and power. They have grand visions, and always see the big picture, which makes them more charismatic. Nevicka and his colleagues have found that leader narcissism has a positive relationship with perceived leader effectiveness [24]. However, current researches have pointed out the dark sides of leader narcissism. For example, Judge et al., indicate that narcissistic leaders constrain by self-knowledge and self-love, lacking empathy, damaging the relationships with followers over time [25]. Rosenthal and Pittinsky also suggest that narcissistic leaders are keen on their own thoughts, pay less attention to followers, and are self-centered that impacts organizational culture [15]. Despite the fact that numerous studies have discussed the bright side and dark side of narcissistic leaders, many of them lack empirical evidence, including the mechanism of how leader narcissism influences followers. Therefore, this study focuses on the relationship between leader narcissism and employee voice behavior, revealing the mechanism of how this happens through empirical research. 

### 2.1. Perceived Leader Narcissism and Employee Voice Behavior

In previous studies, there are few empirical pieces of evidence that have examined the relationship between leader narcissism and employee voice behavior directly, but there is some evidence that indicates the indirect effect of leader narcissism on employee voice behavior. For instance, Campbell and his colleague consider that because narcissistic individuals are overconfident, they love to get praise from others, but are unwilling to accept criticism [19]. Other scholar notes that narcissistic leaders are sensitive to criticism, and they like to adopt knowledge hiding strategies with followers [26]. While other scholar noted that narcissistic leaders are sensitive to others’ evaluation, and also, they are hostile to others’ negative feedback and they challenge negative feedback of themselves [27]. Employee voice behavior makes leaders face a certain challenge to leaders, not only the organization conditions, but also the negative feedback of leadership decisions [28]. When employees perceive the leader’s narcissism, they most likely doubt their openness of leadership and may think leaders will not pay attention to their suggestions, hence the relationship between leader and employee becomes strained. Therefore, employees decide not to take the risk to protect them from the possible revenge from the leader, and they choose not to give feedback to narcissistic leaders. Consistent with previous research, we suggest the following hypothesis:

**Hypothesis** **1.** *leader narcissism negatively relates to employee voice behavior*

### 2.2. Perceived Leader Narcissism and Leader Impression Management

Impression management is defined as an individual’s behavioral modification to maintain the desired perception of themselves in the minds of others [29,30]. Individuals impact on others towards their own perceptions and evaluation in the process of impression management via behavior insinuation, creating a certain image from others [14]. Study has shown that individuals with narcissistic personality trait are adept at processing impression management. For instance, Schnure has found that individuals with a narcissistic personality often get a higher evaluation in job interviews [31]. Brunell and his colleagues have found narcissists are always selected as the group leaders in leaderless group discussion by the evaluation of the manager [32]. Also, the study showed that narcissists usually make a good first impression on people, but with increasing time of interaction, a “false” first impression is likely to be identified. The reason behind impression management is that narcissistic leaders want to get others’ recognition and praise, as well as exploit others [26]. Similarly, narcissistic leaders try to get recognition from followers. This self-impression management behavior comes more likely from the narcissistic personality characteristic, which is motivated by self-serving and self-centered attitude, but unlikely by the pro-social motivation. Because of the motivation of self-serving, narcissistic leaders focus more on their own interests while putting their own interests above others or even teams. Therefore, the pro-social impression management behavior of narcissistic leaders becomes less important for such leaders. On this basis, we thus hypothesize:

**Hypothesis** **2.** *Leader narcissism has a positive relationship with self-serving impression management behavior*

**Hypothesis** **3.** *Leader narcissism has a negative relationship with pro-social impression management behavior*

### 2.3. Leader Impression Management and LMX Quality

The leader-member exchange quality (LMX) indicates the relationship between leader and employee is different from the organizational background and the forms of interpersonal relationships [33]. LMX based on the social exchange theory has pointed out that the relationship form is based on the social exchange process. In this process, both sides account for equality and reciprocity principles [34]. According to LMX theory, if one party has perceived that another party puts out effort in this exchange process, it may persuade this party in future to promote a positive exchange relation quality improvement, which is called positive exchange. On the other hand, if one party has perceived another party pursues only self-interest, it may be perceived unfair or cause hate to hinder or lower the exchange relation quality, which is called negative exchange [35]. Studies have shown that leadership has played an important role in the process of leader-member exchange relationship [36]. Leader behaviors have determined on the evaluation of impression management of leader and attitude by employees, which affect the quality of exchange relationship between them. 

In order to build up a good leader-member exchange relationship, and better governing organization member and management, the leader often does impression management. However, although leader can use impression management to create some “good images” in the eyes of others, the motives perceived by employees behind those images determine employees’ psychological and behavior reaction [37]. Specifically, the motivations perceived by employees behind impression management of leaders may exist for self-serving purposes, rather than pro-social motives. These two different attributions of motives may lead to employees’ variant attitude and psychological behaviors. When employees have perceived leader uses impression management for self-serving purpose, they may think the leader is selfish. Leaders’ impression management may reduce leader-member exchange quality. Adversely, when employees consider leaders’ impression management comes from pro social motive, they may think leaders’ impression management is a positive behavior. Thus, it will enhance the leader-member exchange relationship. We hypothesize the following:

**Hypothesis** **4.** *leaders’ self-serving impression management is negatively related to LMX*

**Hypothesis** **5.** *Leaders’ pro-social impression management is positively related to LMX*

### 2.4. LMX Quality and Employee Voice Behavior

Although studies in the existing literature have examined the direct relationship between LMX quality and employee voice behavior, indirect effect results have shown the quality of LMX could promote employee voice behavior effectively. For example, some scholars posit that LMX quality helps to improve employees’ affective organizational commitment [38]. Moreover, affective organizational commitment would lead employees to consummate organization spontaneously, which promotes organizational development and facilitates employee voice behavior [39]. Also, other scholars indicate that high LMX quality promotes employee organization citizenship behaviors [40]. A meta-analysis has shown that LMX quality is helpful to increase employee organization citizenship behaviors, including employee voice behavior [41]. Based on the above discussion, LMX quality can enhance employee affective commitment, increasing the reciprocal relationship between employee and organization, as well as organizational citizenship behavior [42]. In addition, employee voice behavior is one of the organizational behaviors, which is able to promote the development of the organization. Therefore, we propose the following hypothesis:

**Hypothesis** **6.** *LMX quality is positively related to employee voice behavior*

### 2.5. The Mediating Effect of Leader Impression Management and LMX Quality

The research in leader-member exchange (LMX) usually relied on two theories to explain how LMX varies and develops, that is the role and social exchange theories. People consider low LMX quality relationships are more about economic exchanges and employment contract while considering high LMX quality relationships are beyond the formal work contract, it is more about mutual recognition and support (respect, affect, loyalty) which will motivate follower to gain a higher ability and performance level. Masterson and her colleagues suggest that social exchange theory is closely connected with procedural and interactional justice perceptions in an organization [43]. One common rule of exchange relationship is reciprocity, and this is the base of any social exchange. Beyond purely economic exchange, the social exchange requires two parties involved in a more mutual reciprocal obligation to each other. 

According to above mentioned, narcissistic leaders will present self-interest image more easily which will be considered as self-serving conduct by followers. In this situation, followers may sense more injustice feelings within the dyad relationship, and hence feel bad about the leader-member exchange quality. Narcissistic leaders are more likely to focus on themselves so that there is a lack of social nature in the interaction between leader and follower. The follower may feel less trusted, supported or even respected. As we can infer from the aforementioned, low level of LMX is significantly related to the low level of employee commit and proactive behavior such as organizational citizenship and voice behavior.

Thus, from social exchange theory and the integrative perspective of social exchange theory and justice feeling, we suggest that leader impression management and LMX will mediate the relationship between leader narcissism and follower voice behavior.

## 3. Research Methods

### 3.1. Data Collection and Procedures

We collected data from employees from two state-owned manufacturing company groups (a photoelectric communication enterprise and a machinery processing enterprises) and two private company groups (a biotechnology enterprises and a pharmaceutical enterprises) in China, these company groups all have a nationwide business. The headquarters of these four company groups are located in central China. We sent out all surveys in sealed envelopes to the HR department supervisors of each company groups who have a long-term, high-quality relationship with participants and us, they helped us gather the survey forms according to our instructions. Finally, 300 employees and 73 supervisors completed a supervisor-subordinate paired survey so that some systemic errors such as common method variance can be reduced. and all scales were proceeded with the translate-translate back method by two professors who have published relevant research papers in English to make sure the items can be clearly understood by Chinese workers [44]. In the survey, employees were asked to fill a questionnaire on perceived leader narcissism, pro-social and self- serving impression management, LMX quality, and team leaders were asked to rate their subordinates’ voice behavior. We excluded non-respondent and inappropriate samples (for example, all items rated the same score) and then get 243 employees (with 53% female and 47% male) and 71 supervisors (with 29% female and 71% male) as our valid sample for further analysis. Specifically, 61 participants with 18 leaders were from the photoelectric communication enterprise, 62 participants with 19 leaders were from the machinery processing enterprises, 50 participants with 15 leaders were from the biotechnology enterprises, 70 participants with 19 leaders were from the pharmaceutical enterprises. Overall, for all the employees from four companies, 50.4% of the participants were less than 30 years old, and 49.6% of them were between 30 and 55 years’ old. For their education level, 23% of the participants had education qualification at high school or below, 21% had a junior college education, 46% had a bachelor’s degree and 10% had master’s degree or higher. The employee’s average tenure in the organization was 10.34 years. Among the supervisors, the average age was 37.53 years and 18% of them had master’s degree or higher, 52% had a bachelor’s degree, 16% had a junior college education, and 16% had a high school education or below. The supervisors’ average employment length was 14.65 years and their average employee length in the current company was 6.12 years.

### 3.2. Measurement Scales and Analysis Tools

We adapted well-developed measurement scales and went through the “translate-translate back” procedure. To further check the reliability and validity of every measurement scale, we had run KMO & Bartlett’s test and computed Pattern Matrix, as well as Cronbach’s alpha for the reliability coefficients.

#### 3.2.1. Perceived Leader Narcissism

We adopted a six-item scale from Hochwarter & Thompson [45]. Employees were asked to report their leader narcissism on a five-point Likert type scale (where 1 = “Strongly disagree” and 5 = “Strongly agree”). Items included “My boss is a very self-centered person,’ ‘My boss has an inflated view of him/herself”. To check the reliability coefficients, we computed the Cronbach’s alpha, which is 0.885 for the measurement. KMO & Bartlett’s test is 0.874 (p < 0.001), Pattern Matrix shows that all items are loaded on 1 factor which is good for our study.

#### 3.2.2. Impression Management

We adopted Gardner and Cleavenger’s Leader Impression Management Questionnaire (LIMQ) to measure leader impression management [46]. Fifteen items were used to measure pro-social impression management and 10 items were used to measure self-serving impression management, the details are as follows:

Pro-social impression management was measured using two indicators: a six-item scale measuring Exemplification (e.g., ‘‘is willing to make personal sacrifices for the benefit of others,’’) and a nine-item scale measuring Ingratiation (e.g., ‘‘Makes non-work-related compliments to others,’’). The Cronbach’s alpha = 0.886.

Self-serving impression management was measured using two indicators: a five-item scale measuring Intimidation (e.g., ‘‘Threatens severe sanctions for anyone who defies his or her directives,’’) and a five-item scale measuring Self-Promotion (e.g., ‘‘Points out his or her accomplishments to others,’’). Items were rated on a five-point frequency scale (0 = not at all; 4 = frequently, if not always). The Cronbach’s alpha = 0.892. KMO & Bartlett’s test is 0.916 (p < 0.001) for the pro-social dimension and 0.877 (p < 0.001) for self-serving dimension. Pattern Matrix shows that all items both in pro-social measurement and self-serving measurement are loaded on 2 factors which are consisted of our design.

#### 3.2.3. Leader-Member Exchange

A seven items Likert-type scale was adapted from Graen & Uhl-Bien’s study to measure LMX [16]. Example item includes “I have a good working relationship with my leader.” Items were rated on a five-point frequency scale (see Supplementary Materials). The Cronbach’s alpha = 0.779. KMO & Bartlett’s test is 0.810 (p < 0.001), Pattern Matrix shows that all items are loaded on 2 factor which is consist of the multidimensional nature of LMX studied in Graen & Uhl-Bien’s original paper [16].

#### 3.2.4. Voice Behavior

We adapted Van Dyne and LePine’s six-item scale to measure employee’s general voice behavior [47]. Items were rated on a seven-point Likert scale (where 1 = “Strongly disagree” and 7 = “Strongly agree”), Sample items included “This particular co-worker develops and makes recommendations concerning issues that affect this work group” and “This particular co-worker speaks up and encourages others in this group to get involved in issues that affect the group.”. The Cronbach’s alpha = 0.866. KMO & Bartlett’s test is 0.881 (p < 0.001), Pattern Matrix shows that all items are loaded on 1 factor which is also good for our further analysis.

We used SPSS 25 (IBM, Armonk, NY, USA) and Amos 20 (IBM, Armonk, NY, USA) to process and analyze our data. Specifically, we adapted a standard procedure to test if our hypothesized model is the best fit for structural equation modeling analysis on Amos. As we have five variables in our hypothesized model (five-factor model), we also test four-factor model by combining two measures in to one construct (for example, putting the items of LMX and voice behavior into one variable), as well as three (combining three variables into one), two (combining four variables in to one) and one factor model (combing all variables into one).

## 4. Results

### 4.1. Descriptive Statistics, Validity, and Reliability

Table 1 presents the means, standard deviations, and correlations of all variables. For model fit test and selection, we conducted a series of analysis in which we compared the five-factor model we proposed with other possible constellations such as four-factor model, three-factor model, two-factor model, one-factor model which can be seen in Table 2. We can find that the model fit indexes in the hypothesized five-factor model are significantly better than those in other alternative models. This also means a common method bias will not significantly influence our research results. Thus, all the reliability and validity test, as well as the mode fit test, support our further analysis on the hypothesized five-factor model.

### 4.2. Measurement Model

Table 2 presents multiple indexes in assessing model fit of the measurement model and some comparing models. As shown in the table, the measurement model results indicted the best fit to the data (Five factor model: CMIN = 484.74, DF = 220, CMIN/DF = 2.20, GFI = 0.83, NFI = 0.83, IFI = 0.90, TLI = 0.88, CFI = 0.90, RMSEA = 0.08). These results provided supporting for further examination of the structure model.

### 4.3. Structure Model

**Figure 1:**
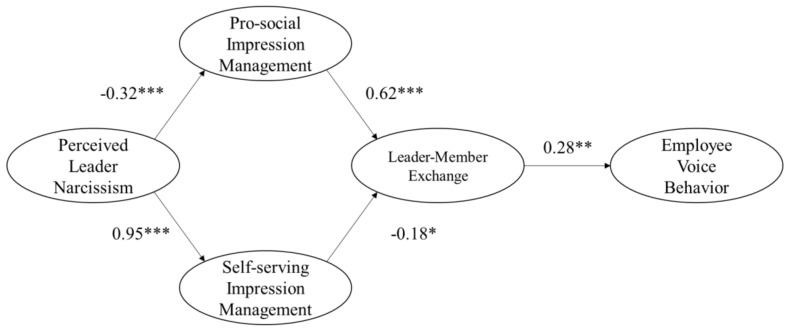
The path coefficient of the hypothesis model. * p < 0.1; ** p < 0.05; *** p < 0.01.

Table 3 presents the summery of the all the model fit indexes. As shown in Table 3, the hypothesized model fit the data better than the alternative models (CMIN = 496.26, DF = 225, CMIN/DF = 2.21, GFI = 0.83, NFI = 0.83, IFI = 0.90, TLI = 0.88, CFI = 0.90, RMSEA = 0.08). The structural model with path coefficients is presented in Figure 1.

Hypotheses 1 and 2 propose that perceived leader narcissism is positively related to leaders’ self-serving impression management and, negatively related to leaders’ pro-social impression management. As shown in Figure 1, the path coefficient of perceived leader narcissistic on self-serving impression management was positive and significant (β = 0.95, p < 0.001). The path coefficient of perceived leader narcissism on pro-social impression management was negative and significant (β = −0.32, p < 0.001). These results supported hypotheses 1 and 2. Hypotheses 3 and 4 propose that leaders’ pro-social impression management is positively related to the quality of leader-member exchange and, leaders’ self-serving impression management negatively related to the quality of leader-member exchange. The path coefficient of self-serving impression management on the leader-member exchange was significantly negative (β = −0.18, p < 0.01) and, the path coefficient of prosaically impression management on the leader-member exchange was significantly positive (β = 0.62, p < 0.001). Hence, hypotheses 3 and 4 were supported. Hypothesis 5 proposes that leader-member exchange is positively related to employee voice, as shown in Figure 1, the path coefficient of leader-member exchange on employee voice was significantly positive (β = 0.28, P < 0.05), indicating that high leader-member exchange would promote employee voice. Therefore, hypothesis 5 was supported.

According to Anderson & Gerbing’s suggestions, we have also examined four alternative models to justify the hypothesized model [48]. In the first alternative model, we tested an indirect effect of perceived leader narcissistic on self-serving impression management via pro-social impression management, because the narcissistic leader is unforgiving [49]. The narcissistic leader may express self-serving impression management by reducing pro-social impression management. But the results indicated that this model does not fit to the data well (CMIN = 632.62, DF = 226, CMIN/DF = 2.80, GFI = 0.79, NFI = 0.77, IFI = 0.84, TLI = 0.82, CFI = 0.84, RMSEA = 0.09). Alternative model 1 was not supported by the data.

Prior studies have identified that the narcissistic leader has a significant impact on the leader’s relationship with the team [50,51]. Therefore, we tested another three alternative models. First, we tested the model that a leader’s perceived narcissism first influences the quality of leader-member exchange, and then, simultaneously decrease pro-social impression management and increase self-serving impression management. Second, we tested the alternative direct relationship between self-serving impression management and pro-social impression management. The model fit results were all presented in Table 3. As shown in the table, none of the three alternative models fit the data well. Comparing the overall model fit of the hypothesized model and all the alternative models, it was suggested that the hypothesized model provide an adequate fit to the data, thus, supporting all the hypotheses further.

## 5. Discussion

In this study, we focus on a social exchange perspective within the relationship between employee perception of leader narcissism, leader impression management, and employee voice behavior. In the empirical test, we examined the chain mediating effect of leader impression management and LMX had on the relationship between leader narcissism and employee voice behavior. The results show that employee perception of leader narcissism is negatively related to their perception of leader pro-social behavior and positively related to self-serving behavior. And these two impression management behavior will have a different impact on leader-member exchange quality which will then positively influence employee voice behavior. In our data analysis, the results indicate that leader narcissism is more related to self-serving conduct perception, which will negatively affect the employee voice behavior through the mediating effect of LMX quality. The result has shown that narcissistic leader reduces pro-social impression management behavior, but increases self-serving impression management behavior, thus lower the quality of LMX which result in less employee voice behavior. The results especially the main effect of leader narcissism reduce employee voice behavior is consistent with Liu and his colleagues’ study [12]. This research provides several theoretical contributions in several aspects: First, this paper provides more empirical evidence to the relationship between leader narcissism and employee voice behavior from the perspective of impression management, which has filled the gap in the existing literature. Although some studies in the past have mentioned the possible connection of narcissistic leader and employee voice behavior, these have not been fully discussed [15]. Second, this research based on the social exchange theory has revealed the mechanism of leader narcissism and employee voice behavior. Most literatures have shown the antecedent factors of employee voice behavior from the perspective of “risk” [6], which are rational. This paper based on social exchange theory has considered a leader in associate with employees in the process of reciprocity and emotional mechanism on the effect of employee voice behavior, which is an important complement of existing literature. In addition, this research has studied the link between leader narcissism and LMX quality, from the perspective of impression management. Although previous studies have mentioned that leader narcissism would worsen the relationship between leader and employees, the mechanism lack empirical tests. This study has answered this question of how a leader’s narcissism influences LMX quality, revealing this mechanism from the perspective of impression management.

This study has several practical implications: First, this study empirically proved that leader narcissism has an inhibitory effect on employee voice behavior. To promote organizational development and improvement, the team leader should show humility, such as how to objectively view the value of employees and avoid the negative effect of narcissism that brings to the organization. Second, this research has shown the impact of leadership style on employee behavior and the intrinsic motivation of attribution of leaders’ impression management. When leader behavior is attributed to pro-social motivation, it enhances the relationship of LMX quality; In contrast, when leader behavior is attributed to self-serving motivation, it damages the relationship of LMX quality. Therefore, the leader should not only carry on impression management but also should pay more attention to the attribution management of behavior and motivation. For example, as a team leader, he or she should do some pro-social behavior to build a better image and enhance member exchange relationship, such as helping employees to solve problems, helping employees for career development, listening to employees from their perspective, etc. Moreover, this study has also found employee voice behavior not only depends on the potential risk they may feel but also the reciprocity and emotional relationship of leader and employee. This suggests that employee has both a rational side and the emotional side. For the development of the organization, leaders should create an atmosphere to encourage employees to speak up, and they should also consider the effect of intrinsic motivation of employee voice behavior. It’s better for the leaders to talk with employees sentimentally and rationally.

Although this study makes a certain theoretical contribution and has practical implications, our research also has several limitations that need to be explored in future research. First, although this study used leader-employee dyadic data to avoid the common method bias problem, it used a cross-sectional design. Because we did not test the longitudinal study, thus the result has not shown the causality, especially the effect of leader humility on employee voice behavior. Hence, future studies may consider longitudinal design or experiment study to test its further relations. Second, this paper may not take some of the boundary factors into consideration about the effect of leader narcissism and employee voice behavior, such as other personality traits, or organization culture, etc. Different employees may react differently towards what leader narcissism may bring out of them; under a different organizational culture, the effect of leader narcissism on employee voice behavior may also be different. Future studies need to take the personality traits of employees and organizational culture into account, testing the variation of leader narcissism on employee voice behavior under different circumstances. Finally, this study has not controlled the key mechanism mentioned, which are the effect and risk that impact on employee voice behavior in past literature. Due to the lack of specific scales, this paper distinguishes from the theory and logic, the future study may consider developing a relative scale to differentiate various mechanism of the impact on employee voice behavior.

## 6. Conclusions

The purpose of this paper was to examine the role of leader impression management and its influence on leader-member exchange quality as well as employee voice behavior. We found that leader narcissism is negatively related to leader pro-social impression management but positively related to leader self-serving impression management. And the correlation between leader narcissism and self-serving impression management is more significant than that of leader pro-social impression management. The results show that narcissistic leaders will reduce employees’ general voice behavior through self-serving conduct perception and reduced LMX quality. This finding answered the question of how a leader’s narcissism influences LMX quality as well as employee voice behavior, revealing this mechanism from the perspective of impression management.

## Figures and Tables

**Table 1:** Means, Standard Deviations, and Correlations.

Variables	Mean	SD	1	2	3	4	5	6	7	8
Employee gender										
Employee Age	32.27	7.430	0.17 *							
Employee Education	2.41	0.965	0.02	−0.37 *						
Employee Tenure	10.45	8.189	0.18 **	0.94 *	−0.50 **					
Perceived leader narcissistic	2.047	0.701	0.09	0.05	−0.13	0.08				
Pro-social impression management	3.729	0.551	0.07	−0.14 *	0.23 **	−0.17 *	−0.38 **			
Self-serving impression management	2.261	0.701	0.06	0.06	−0.08	0.07	0.75 **	−0.39 **		
Leader member exchange	3.572	0.501	0.04	−0.15 *	0.25 **	−0.16	−0.38 **	0.54 **	−0.33 **	
Employee voice	5.091	0.882	0.03	−0.09	0.16 *	−0.15	−0.15 *	0.16 *	−0.17 *	0.13

* p < 0.05; **p < 0.01, SD: Standard Deviation.

**Table 2:** Results of measurement model and comparing models.

Models	CMIN	DF	CMIN/DF	GFI	NFI	IFI	TLI	CFI	RMSEA
Measurement model (Five factor model)	484.74	220	2.20	0.83	0.83	0.90	0.88	0.90	0.08
Four factor model: Self-serving management + Pro-social impression management	629.10	224	2.80	0.79	0.78	0.85	0.82	0.84	0.09
Four factor model: Perceived leader narcissistic + self-serving impression management	502.90	224	2.25	0.83	0.82	0.89	0.88	0.89	0.08
Four factor model: Perceived leader narcissistic + pro-social impression management	638.71	224	2.85	0.79	0.78	0.84	0.82	0.84	0.09
Four factor model: leader member exchange + pro-social impression management	578.32	224	2.58	0.81	0.80	0.86	0.85	0.86	0.09
Three factor model: Leader member exchange + self-serving impression management + pro-social impression management	791.38	227	3.49	0.74	0.72	0.78	0.76	0.78	0.11
Three factor model: Perceived leader narcissistic + self-serving impression management + pro-social impression management	653.73	227	2.88	0.79	0.77	0.84	0.82	0.84	0.09
Four factor model: Leader member exchange + perceived leader narcissistic + Self-serving impression management + pro-social impression management	934.98	229	4.08	0.67	0.67	0.73	0.69	0.73	0.12
Five factor model: Voice + leader member exchange + Perceived leader narcissistic + Self-serving impression management+ pro-social impression management	1527.32	230	6.64	0.53	0.46	0.50	0.49	0.50	0.16
Independence model	2836.98	253	11.21						

CMIN: Chi-square; DF: Degree of Freedom; CMIN/DF: Chi-square to DF Ratio; GFI: Goodness of Fit index; NFI: Normed Fit Index; IFI: Incremental Fit Index; TLI: Tucker-Lewis Index; CFI: Comparative Fit Index; RMSEA: Root Mean Square Error of Approximation.

**Table 3:** Results of structure model and comparing models.

Alternative Models	CMIN	DF	CMIN/DF	GFI	NFI	IFI	TLI	CFI	RMSEA
Hypothesis model	496.26	225	2.21	0.83	0.83	0.90	0.88	0.90	0.08
Alternative Model 1: ln-pro-self-lmx-voice	632.62	226	2.80	0.79	0.77	0.84	0.82	0.84	0.09
Alternative Model 2: ln-lmx- (self, pro) -voice	651.31	225	2.90	0.79	0.77	0.84	0.81	0.84	0.09
Alternative Model 3: ln-lmx-self-pro-voice	667.98	226	2.96	0.79	0.77	0.83	0.81	0.83	0.10
Alternative Model 4: ln-lmx-pro-self-voice	651.57	226	2.88	0.80	0.77	0.84	0.82	0.84	0.09

CMIN: Chi-square; DF: Degree of Freedom; CMIN/DF: Chi-square to DF Ratio; GFI: Goodness of Fit index; NFI: Normed Fit Index; IFI: Incremental Fit Index; TLI: Tucker-Lewis Index; CFI: Comparative Fit Index; RMSEA: Root Mean Square Error of Approximation.

## Supplementary Materials

The following are available online at https://www.mdpi.com/1660-4601/16/10/1819/s1, Scales for measurement.
