# Supplementary material for: How Does Leader Narcissism Influence Employee Voice: The Attribution of Leader Impression Management and Leader-Member Exchange
Source: Int J Environ Res Public Health. 2019 May 22;16(10):1819. doi: 10.3390/ijerph16101819 (PMC6571719; doi:10.3390/ijerph16101819)
Supplement: Supplementary file 1 [file ijerph-16-01819-s001.pdf]

Appendix

Scales for measurement

Perceived Leader Narcissism:

1. My boss is a very self-centered person.
2. My boss has an inflated view of him/herself.
3. My boss brags about him/herself to get positive strokes from others.
4. My boss will do one favor as long as he/she gets two or more in return.
5. My boss will go out of his/her way to cause me harm to get ahead.
6. My boss always has to be the center of attention no matter what.

Impression Management:

Pro-social impression management

1. Presents himself or herself as warm and charming to others.
2. Behaves in ways that are consistent with others' expectations of him or her.
3. Describes himself or herself in ways that others will find personally appealing.
4. Inquires about my nonwork life (for example, asking about my family).
5. Praises other's ideas or work.
6. Outwardly expresses agreement with others' opinions, even though he or she may really disagree.
7. Makes non-work-related compliments to others.
8. Offers to do things for others, even though he or she is not required to do so; that is, does personal favors for others.
9. Flatters others regarding their appearance, conduct, and so on.
10. Leads by example whenever possible.
11. Is generous with his or her time and energy in helping others.
12. Is willing to make personal sacrifices for the benefit of others.
13. Holds his or her performance up to high standards.
14. Demonstrates a high level of personal integrity.

Self-serving impression management

1. Makes it clear that his or her decisions are to be followed without question.
2. Threatens severe sanctions for anyone who defies his or her directives.
3. Demands respect from his or her subordinates.
4. Publicly ridicules persons who oppose him or her.
5. Makes threats to persons who do not meet his or her expectations.
6. Points out his or her accomplishments to others.
7. Boasts about his or her achievements to others.
8. Takes advantage of opportunities to demonstrate his or her skills and abilities.
9. Readily takes credit for past and current successes.
10. Uses status symbols to communicate his or her position or power.

LMX measure:

1. Do you know where you stand with your leader…do you usually know how satisfied your leader is with what you do? (Does your member usually know)

Rarely Occasionally Sometimes Fairly Often Very Often

2. How well does your leader understand your job problems and needs? (How well do you understand)

Not a Bit A Little A Fair Amount Quite a Bit A Great Deal

3. How well does your leader recognize your potential? (How well do you recognize)

Not at All A Little Moderately Mostly Fully

4. Regardless of how much formal authority he/she has built into his/her position, what are the chances that your leader would use his/her power to help you solve problems in your work? (What are the changes that you would)

None Small Moderate High Very High

5. Again, regardless of the amount of formal authority your leader has, what are the chances that he/she would "bail you out," at his/her expense? (What are the chances that you would)

None Small Moderate High Very High

6. I have enough confidence in my leader that I would defend and justify his/her decision if he/she were not present to do so? (Your member would)

Strongly Disagree Disagree Neutral Agree Strongly Agree

7. How would you characterize your working relationship with your leader? (Your member)

Extremely Ineffective	Worse Than Average	Average	Better Than Average	Extremely Effective

Voice measure:

1. This particular co-worker develops and makes recommendations concerning issues that affect this work group.
2. This particular co-worker speaks up and encourages others in this group to get involved in issues that affect the group.
3. This particular co-worker communicates his/her opinions about work issues to others in this group even if his/her opinion is different and others in the group disagree with him/her.
4. This particular co-worker keeps well informed about issues where his/her opinion might be useful to this work group.
5. This particular co-worker gets involved in issues that affect the quality of work life here
6. This particular co-worker speaks up in this group with ideas for new projects or changes in procedures.
